# Halogen-Free Flame-Retardant Compounds. Thermal Decomposition and Flammability Behavior for Alternative Polyethylene Grades

**DOI:** 10.3390/polym11091479

**Published:** 2019-09-10

**Authors:** Adriaan Stephanus Luyt, Sarah Shahid Malik, Soumia Abderrazak Gasmi, Athanasios Porfyris, Anna Andronopoulou, Dimitrios Korres, Stamatina Vouyiouka, Michael Grosshauser, Rudolf Pfaendner, Robert Brüll, Constantine Papaspyrides

**Affiliations:** 1 Center for Advanced Materials (CAM), Qatar University, P.O. Box 2713, Doha, Qatar; 2 Laboratory of Polymer Technology, School of Chemical Engineering, National Technical University of Athens, Zographou Campus, 157 80 Athens, Greece; 3 Fraunhofer Institute for Structural Durability and System Reliability LBF, Schlossgartenstr. 6, 64289 Darmstadt, Germany

**Keywords:** halogen-free flame retardants, low-density polyethylene, linear low-density polyethylene, intumescence, thermal decomposition, flammability, Kissinger model, Coats-Redfern model

## Abstract

The effect of six halogen-free flame retardant (FR) formulations was investigated on the thermal stability of two low-density polyethylenes (LDPE) and one linear low-density polyethylene (LLDPE), by means of thermogravimetric analysis (TGA) under nitrogen and air atmosphere. The relative data were combined with flammability properties and the overall performance of the FRs was correlated with the type of branching in the polyethylene grades and to their processing behavior. The thermal degradation kinetics was further determined based on the Kissinger and Coats-Redfern methods. In terms of flammability, the addition of a triazine derivative and ammonium polyphosphate at a loading of 35 wt. %. was found to be the most efficient, leading to UL 94 V0 ranking in the case of the LDPE grade produced in an autoclave reactor.

## 1. Introduction

Polyethylenes (PEs) are the most widely used commodity polymers with a high potential of value-adding via proper formulation development. Increasing amounts of PEs are nowadays used in electrical appliances, wires and cables, building pipes, roofing, etc., because of their mechanical durability, good chemical resistance, low density, no toxicity, good electrical insulation and excellent processability [1]. The relevant applications need to be flame retarded in order to comply with stringent fire safety standards of the finished products. However, PEs are among the most flammable materials with high heat of combustion, low limited oxygen index (LOI) and high heat release, leaving little or no residual char. To improve the flame resistance, halogen-containing flame retardants (HFRs), such as various brominated FRs (decabromodiphenyl ether, tetrabromobisphenol A, tris(tribromoneopentyl) phosphate) are mainly used in combination with antimony oxide [2,3]. Nevertheless, HFRs present significant disadvantages, namely corrosion of the equipment during processing, production of toxic gases and smoke in the case of fire as well as environmental challenges. 

Halogenated flame retardants are therefore often phased out and replaced with halogen-free alternatives [4,5]. Among the alternatives, inorganic flame retardants, such as aluminum hydroxide and magnesium hydroxide, need to be added to a polyethylene in high loadings (>50 wt. %) in order to pass various fire standards tests. These high levels impair negatively on the mechanical, physical and rheological properties, as well as the processing ability of the polyolefins [2]. On the other hand, phosphorus– and nitrogen-based compounds, including intumescent flame retardant systems (IFRs), present a viable alternative. IFRs play an effective role, mainly through a condensed-phase mechanism forming a carbonaceous foam residue (swollen char) on the surface of the polymer that acts as a heat insulator and physical barrier to the transport of oxygen and pyrolysis products. Commonly used IFRs consist of three main ingredients, namely an acid source, a carbon source (char forming agent, CFA) and a gas source (blowing/spumific agent). The acid source decomposes at a low temperature and generates inorganic acid, such as phosphoric acid, polyphosphoric acid and metaphosphoric acid. An esterification reaction takes place between the inorganic acid and the carbon source, and the carbon source turns into the protective char layer after the dehydration temperature. The blowing agent releases gases (e.g., water vapor, NH_3_) causing the ester to create a foam that forms an insulating barrier which adheres to the substrate. Finally, the ester decomposes to form a tough carbon matrix acting as a shield for the polymer [6]. In each case, the ratio of acid source, charring source and blowing agent should be optimized per polymer type.

A widely used and effective IFR system is based on ammonium polyphosphate (APP), pentaerythritol (PER) and melamine (MA). It is generally agreed that APP, a precursor of polyphosphoric acid, promotes the acid hydrolytic reaction of the substrates, while it also serves as a blowing agent [1,7,8,9,10]. In addition, new CFAs, i.e., triazines and derivatives, have attracted more and more attention; they are good carbon sources and potential gas sources, because of their abundant nitrogen and structure of tertiary nitrogen, presenting also water resistance [1,7,9]. Therefore, the objective of the current paper was to investigate the effect of commercially available flame retardants on the thermal stability and flammability properties (vertical burning test, UL 94V) of low-density polyethylene (LDPE) and linear low-density polyethylene (LLDPE) grades. The applied formulations involved a nitrogen-based compound (FR1), two intumescent systems with triazine derivatives (FR2, FR3), a commercial blend of phosphorus-nitrogen compounds (FR4) and two intumescent systems with pentaerythritol derivatives (FR5, FR6). Single phosphorus-based FRs were not examined since it is well-known that they mainly are efficient in oxygen-containing polymers, such as polyamides: they can produce derivatives with strong dehydration and when oxygen is provided the derivatives can dehydrogenate the substrate to increase the amount of residue after combustion [11].

**Figure 1:**
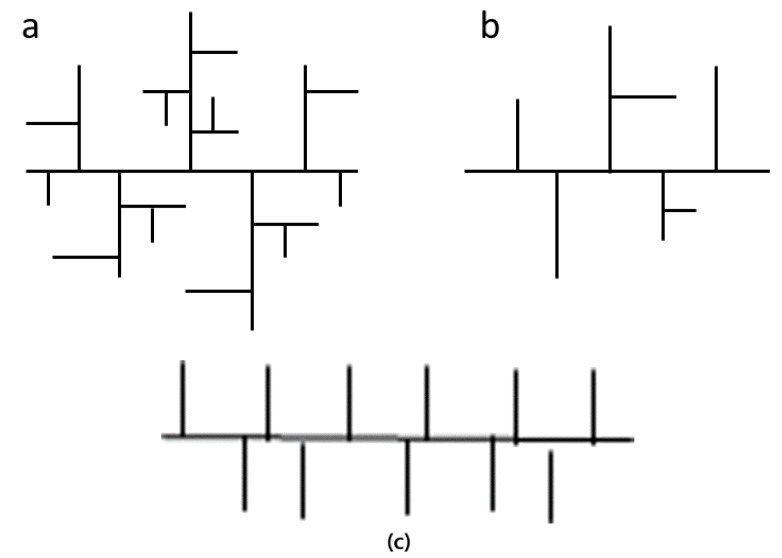
(**a**) Autoclave product (LDPE-A), (**b**) tubular product (LDPE-T) both of molecular weight ≈ 5 × 10^5^ g mol^−1^ [12,13,14], (**c**) linear low-density polyethylene (LLDPE) [15].

The overall performance of the FR formulations was herein correlated with the type of branching in the polyethylene grades and to their processing behavior, a study which is being performed for the first time, since one PE type is usually studied in pertinent works. More specifically, two types of LDPE were examined: the LDPE-A is manufactured in an autoclave (batch process) and the LDPE-T in a tubular reactor (continuous process), and they differ in the type and level of long-chain branching and in the molecular shape (Figure 1). LDPE-A is produced at a constant temperature and under practically ideal mixing and presents short- and long-chain branching points distributed essentially at random along the chains irrespective of molecular weight. Its molecules show tree-like branching and a nearly globular shape. The LDPE-T is produced under variable conditions (not a constant temperature) and shows a narrower molecular weight distribution, but wider distributions of long- and short-chain branching. In fact, the LDPE-T displays less long- and short-chain branching with growing molecular weight and the molecules are characterized by comb-like long-chain branching and consequently have more extended (rod-like but flexible) conformations [12,13,14]. On the other hand, the LLDPE is a copolymer of ethylene and *α*-olefins, presenting narrower molecular weight distribution and not containing long-chain branching compared to the LDPE grades [15]. Finally, overall characterization including melt flow rate (MFR), mechanical properties and melting behavior (DSC) of the most promising FR compounds were performed so as to evaluate the viability of the relevant FR systems per polyethylene type.

## 2. Materials and Methods

### 2.1. Starting Materials

Two commercial low-density polyethylene grades (LDPE-A, LDPE-T, MFR = 0.3 g/10 min) and one linear low-density polyethylene (LLDPE, MFR = 1 g/10 min, co-monomer 1-butene) were provided by Qatar Petrochemical Company (QAPCO, Doha, Qatar). LDPE-A refers to the LDPE manufactured in the autoclave (batch process) and LDPE-T to the one manufactured in the tubular reactor (continuous process). The weight average molar masses and disparities of the samples were characterized by gel permeation chromatography with multiangle laser light scattering (HT-GPC-MALS) and found to be *M*_w_ = 334.4 kg mol^−1^/Ð = 4.4 (LDPE-A), *M*_w_ = 155.1 kg mol^−1^/Ð = 2.8 (LDPE-T) and *M*_w_ = 117.9 kg mol^−1^/Ð = 2.1 for LLDPE. Their long-chain branching (LCB) content was similarly characterized and LDPE-A was found to contain significantly more LCB than LDPE-T, whereas LLDPE was found to be mostly linear. This is evident from the plots of the radius of gyration (*R*_g_) of the samples as a function of molar mass (conformation plots) shown in the Supplementary Information (Figure S1). The conformation plot for a linear polymer shows a slope of about 0.57, and with increasing LCB content smaller slopes are found as the molecules become more compact. LDPE-A is therefore much more compact than LDPE-T.

The examined halogen-free flame retardants (FRs) were provided by BASF (Berlin, Germany), MCA Technologies GmbH (Biel-Benken, Switzerland), Clariant (Knapsack, Germany) and Perstorp (Perstorp, Sweden) (Table S1).

### 2.2. Preparation of Flame Retarded Compounds

The halogen-free FR systems were examined in a total content of 5–35 wt.% (Table 1) with ratios of the char-forming agent to the acid source (CFA:APP) equal to 1:3 and 1:1.5. The incorporation was performed in a twin-screw extruder (extruder-Lab compounder, KETSE 20/40 EC, model 838106, 170–200 °C, 70 rpm) following a dry mixing stage. The compounded material was then injection molded (Arburg All-rounder 570 C golden edition, 180–215 °C) to prepare specimens for UL94 with dimensions 127 mm × 12.7 mm × 1.6 mm, impact test specimens with dimensions of 120 mm × 12.6 mm × 3 mm, and dumbbell shaped specimens with dimensions 160 mm × 13 mm × 3 mm. 

### 2.3. Characterization 

#### 2.3.1. HT-GPC-MALS

HT-GPC-MALS characterizations were performed using an Agilent PL 220 oven (Agilent Technologies, Frankfurt, Germany) equipped with a PolymerChar IR4 detector (Polymer Char, Valencia, Spain) for concentration detection and a Wyatt Heleos Dawn II MALS (WYATT Technology Europe GmbH, Dernbach, Germany) for direct determination of molar masses and LCB content. The separations were performed at 150 °C in 1,2,4-trichlorobenzene (containing 1 g butylated hydroxytoluene/L for stabilization) using three Agilent PLgel Olexis (300 × 7.5 mm (L × I.D.)) columns (Agilent Technologies, Frankfurt, Germany). 200 µL polymer solution were injected per analysis.

#### 2.3.2. SEM 

Scanning electron microscopy (SEM) was performed on the surfaces of the injection molded specimens in a FEI Quanta 200 electron microscope (Thermo Fischer SCIENTIFIC, Hillsboro, USA) at an accelerating voltage of 2–5 kV. The samples were sputter gold coated for 30 s using an Agar sputter coater. 

#### 2.3.3. UL 94 V Testing

The flammability was assessed according to UL 94 vertical burning tests (ASTM D3801) on injection molded bars, following a pretreatment of the specimens for 45 h at 23 °C and 50% RH. 

#### 2.3.4. Thermogravimetric Analysis (TGA)

The thermal decomposition was studied via thermogravimetric analysis in a Mettler Toledo TGA/DSC 1 HT instrument (Mettler-Toledo GmbH, Greifensee, Switzerland). Approximately 15–30 mg of sample was heated from 30 to 800 °C at 10 °C min^−1^ under air (thermo-oxidative decomposition) and nitrogen (thermal decomposition) atmospheres, while additional heating rates (5, 15, 20, 25 °C min^−1^) were examined under air atmosphere. The onset of decomposition temperature was defined as the temperature at 5% weight loss (*T*_*d*,5%_), the degradation temperature (*T_d_*) was determined at the maximum rate of weight loss, and the char yield as the % residue at 600 and 800 °C. The pure FR additives, pure polymers and the relevant FR-containing compounds were accordingly analyzed.

#### 2.3.5. Differential Scanning Calorimetry (DSC)

DSC analysis was performed using a Mettler DSC 1 STARe system (Mettler-Toledo GmbH, Greifensee, Switzerland) under nitrogen flow (20 mL min^−1^). Approximately 15 mg of each polyolefin sample was placed in a 40 μL aluminum crucible and was initially heated from 30 to 160 °C (1st heating) at a heating rate of 10 °C min^−1^, then cooled at the same rate to 30 °C (cooling) and reheated to 160 °C at the same rate (2nd heating). The crystallization point (*T*_c_) was obtained from the DSC cooling cycle. The melting point and enthalpy of fusion were derived from the second heating cycle, and the mass fraction crystallinity (*x_c_*, %) was computed according to Equation (1):

(1) $$x_{c}=100\times \frac{\Delta H_{f}}{\Delta H_{0}(1-\phi )}$$

where, Δ*H_f_* is the heat of fusion (J g^−1^) and Δ*H*_0_ is the heat of fusion of 100% crystalline polymer (J g^−1^), *φ* is the additive nominal mass fraction in the compound. The value for Δ*H*_0_ was 293 J g^−1^ [16].

#### 2.3.6. Melt Flow Rate (MFR)

The melt flow rate (MFR, g/10 min) was measured at 190 °C and 2.160 kg, according to ASTM D1238, using a Dynisco model 4004 capillary rheometer (Dynisco Europe GmbH, Heilbronn, Germany).

#### 2.3.7. Mechanical Properties

The tensile testing was performed at an elongation rate of 10 mm min^−1^ using a Lloyd LR50K Plus universal testing machine (Lloyd Instruments Ltd, West Sussex, UK). The gauge length was 50 mm. The Young’s modulus (E) was manually calculated from the slope of the stress-strain curve between strain values of 0.2 and 2.2%. The impact test specimens were cut down to a length of 63.5 mm and were notched in the center (45° notch and 2 mm depth) so as to meet the ASTM D256 definitions. Impact tests were performed in an Instron Wolpert PW5 impact testing apparatus (Instron, Norwood, USA) and the Izod impact strength (*a_iN_* in kJ m^−2^) was calculated according to Equation (2), where *E_c_* is the corrected measured absorbed energy during impact in J, *h* is the thickness of the tested specimen in mm and *b_N_* is the remaining width of the tested specimen in mm. For each measurement five specimens were tested.

(2) $$a_{iN}=\;\frac{E_{c}}{h\;\times b_{N}}\;\times \;10^{3}$$

## 3. Results and Discussion

### 3.1. Thermal Decomposition of FR-Containing Compounds

Thermogravimetric analysis (TGA) is a common technique to evaluate the thermal stability of various polymers giving information on weight loss, but no chemical information [17,18]. In our work, the thermal decomposition profiles of the pure components (additives, pure polymers) and of the FR-containing compounds were examined at a heating rate of 10 °C min^−1^ under air and nitrogen atmospheres, and the relevant data correlated with the flammability properties (UL94 V results). 

Starting with the pure polymers, all three grades exhibited a one-step decomposition (Figure S2a) with the onset of the polyethylene backbone degradation in the range of (419–448) ± 3 °C (*T*_*d*,5%_) under nitrogen (Figure S2b). The maximum rate of weight loss was observed at 460 ± 8 °C and 466 ± 4 °C for LDPE-A and LDPE-T respectively, while for LLDPE at a higher value of 476 ± 1 °C. All the grades had negligible residue values, verifying the little char formation which characterizes polyolefins. When comparing the PE grades, LLDPE presented a higher thermal stability under nitrogen than LDPE-T, which in turn was found more stable than LDPE-A. The higher thermal stability of LLDPE when compared to the LDPE grades is in agreement with literature [16,19], and this ranking can be correlated with the branching degree and the number of the tertiary carbons that are the most reactive parts of the polymer molecule. LLDPE presents minimal branching when compared to the LDPE grades, and thus higher thermal stability. 

On the other hand, the lower thermal stability of LDPE-A can be attributed to a larger number of tertiary carbons compared to LDPE-T. The same trend was observed in the *T_d_* values during the thermo-oxidative degradation (TGA under air), but for the onset of degradation (*T*_*d*,5%_), the temperatures were found to be similar, independent of the polyethylene type (410–417 °C). Furthermore, as anticipated, pure thermal degradation (under nitrogen) occurred at higher temperatures than thermo-oxidative degradation.

Turning to the pure FR additives, TGA analysis under nitrogen revealed that the *T*_*d*,5%_ varied between 243 and 380 °C, which is significantly lower than those of the pure polyethylene grades (Figure S3). More specifically, the degradation of the alkoxy amine (triazine derivative, Flamestab NOR116), which is a nitrogen-based FR, was found to be a two-step process with maximum rates of weight loss at 290 and 437 °C, and with a residue of 4% at 800 °C, implying a low charring ability. PPM Triazine HF is also a nitrogen-based additive but it presents a higher residue (17.3% under N_2_), showing its role as a charring source. The commercial mixture of the Triazine HF with ammonium polyphosphate (PPM Triazine 765) exhibits negligible char at 800 °C with more than two steps of thermal decomposition. The latter can be attributed to the used ratio of the CFA:APP in the commercial system. When the CFA content is high, the corresponding system has a superfluous charring source and a lack of acid source, so part of the CFA cannot be dehydrated into char and decomposes into gas products [11].

Similarly, the two pentaerythritol derivatives (Charmor PP100 (polypentaerythritol) and Charmor DP40 (dipentaerythritol)) did not yield a significant amount of char (3.6%, 3.9%), and they presented mainly one step of thermal degradation with respective onsets of degradation at 243.1 °C and 303.8 °C. The pentaerythritol derivatives obviously decompose into gas phase products in the absence of an acid source. As far as ammonium polyphosphate (Exolit AP422) is concerned, its degradation profile presented a first weight loss peak at ca. 330 °C under N_2_, attributed to the elimination of NH_3_ and H_2_O during the thermal decomposition of the polyphosphate. The second peak appearing at 641 °C is attributed to the release of phosphoric acid, polyphosphoric acid and metaphosphoric acid with the decomposition of APP [11]. Finally, the char residue is high, i.e., 23.5% at 800 °C, because during the course of its decomposition, polyphosphoric acid itself is reverted to phosphoric acid and volatile gases, such as NH_3_ and N_2_, which cause the char to swell. NH_3_ reacts with phosphoric acid, gathering in the swollen char layer to produce the corresponding salt, and this salt constitutes a block protecting the underlying material [9]. 

ADK Stab FP 2200 exhibited the most promising TGA curve in terms of intumescence. It is a blend of phosphorus-nitrogen compounds and the thermal degradation occurs in two main steps, with the first *T_d_* at 292 °C and the second at 412 °C, corresponding potentially to the loss of water and small molecules such as NH_3_, further carbonization and the thermal degradation of char residue. The TGA curve is similar to the one of the alkoxy amine (Flamestab NOR116), but the char residue at 800 °C was found to be much higher, i.e., 51%. This char yield was in fact the highest value among all the examined FRs, implying that the pertinent system combines a carbon source, an acid source and a blowing agent, and presents high charring ability under the synergism of phosphorus and nitrogen compounds.

**Figure 2:**
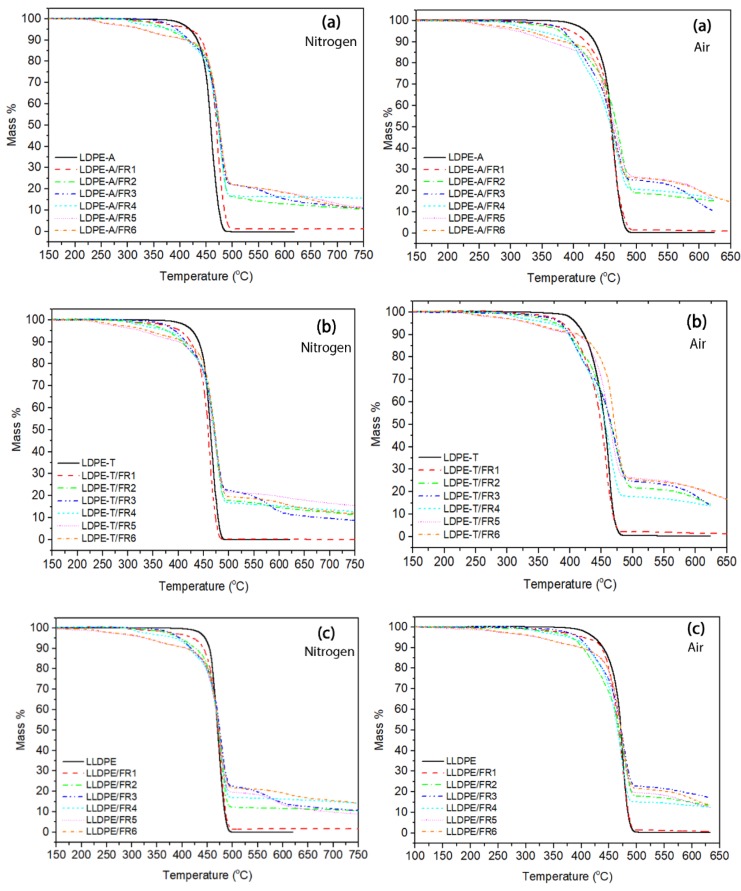
TGA curves under nitrogen and air of FR-containing compounds (**a**) LDPE-A, (**b**) LDPE-T, (**c**) LLDPE.

The presence of the FRs in the polymer matrix resulted in changes in the TGA curves compared to the reference polymers. As can be seen in Figure 2, the thermal decomposition of the FR-containing compounds involved two or even more degradation steps, with the onset temperature lower compared to the onset of the pure grades; the *T*_*d*,5%_ value was lower in the range of 330–422 °C under nitrogen (vs. 419–448 °C for pure polymers), with the standard deviation of the mean (STDEV) ranging from 1–5 °C for the FR3 and FR4 compounds. This decrease is attributed to the lower stability of the bonds in the FR molecules (such as P–O and C–N) compared to the uniform C–C bonds in polyethylene, and thus the earlier degradation of the additives [6,20]. The lowest *T*_*d*,5%_ values were observed in the cases of FR5 and FR6, i.e., when the pentaerythritol derivatives and APP were used, and can be attributed to the formation of thermally unstable ester mixtures between the –P–OH group in the APP molecules and the –OH group in the CFA [8]. 

When comparing the degradation temperatures at the maximum weight loss (*T_d_*), the addition of FRs resulted in an increase in *T_d_*, proving the formation of a protective char layer and improving thus the thermal stability of the grades. In the work of Makhlouf et al. [20], an increase in the *T_d_* by 30 °C was observed in the case of the effective LLDPE FR formulation. In the work of Xie and Qu [21], the flame retarding mechanism of the expandable graphite/halogen-free FR system for LLDPE also gave rise to such an increase in the thermo-oxidative degradation temperature. In our work, as can be seen in Figure S4 in the Supplementary Material, the *T_d_* increase is kept at lower values: for the two LDPE grades, *T_d_* reached 478 °C (STDEV: 1–3 °C) vs. 460 and 465 °C (ca.15 °C increase), while for the LLDPE compounds the increase was almost insignificant (up to 2 °C). The trend was found similar under thermo-oxidative degradation (Figure S4b), with the difference that the temperatures (*T*_*d*,5%_, *T_d_*) were shifted to lower values under air, as was the case with the pure polymers.

**Figure 3:**
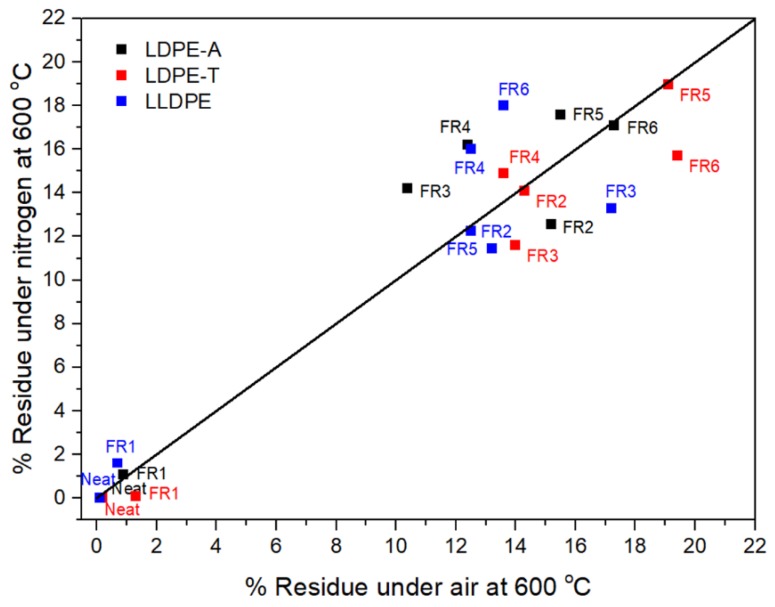
TGA residue (%) under nitrogen and air atmosphere for the FR-containing compounds.

The residue in the TGA can also be correlated with the formation of a protective char layer during the polymer combustion [1,7,8,11,20,22,23] (Figure 3). FR1, which contained a nitrogen-based additive, failed to increase the char yield, so it is anticipated that the relevant formulation will not be efficient due to a lack of the acid source. On the other hand, the addition of the other FRs dramatically increased the residue under air and nitrogen atmospheres, reaching almost 19% under nitrogen at 800 °C, with the highest value observed for FR4 (16.2 ± 0.4%) for LDPE-A, FR5 (19%) for LDPE-T, and FR6 (18%) for LLDPE. It is important to emphasize that the low residue of the pure pentaerythritol derivatives (Charmor DP40 and PP100) was not observed in the FR5 and FR6 formulations, since along with the acid source, they can result in an effective intumescent system. However, it should be pointed out that in the literature, there is no direct correlation between the char residue and the UL94 V ranking; when the compound presents higher residue, it presents potentially better flammability properties, but it is not necessary that the highest amount of char gives V0 in UL94 ranking. This also has to do with the proper ratio of charring agent to acid source, so as to produce the optimum char layer morphology, e.g., tiny bubbles embedded on the surface of each large bubble, and a compact and continuous intumescent char [1].

### 3.2. Kinetics of Thermal Decomposition

The Kissinger method was used to study the kinetics of the thermal decomposition of the FR polyolefin systems under air atmosphere. The Kissinger method (Equation (3)) relates the apparent activation energy of the solid reaction to the logarithm of the heating rate (*q*) and the reciprocal of the absolute temperature at the maximum degradation rate (*T_d_*) [24,25,26]:

(3) $$\ln\left(\frac{q}{T_{d}^{2}}\right)=\left\{ln\frac{AR}{E_{a}}+\ln\left[n{\left(1-a_{d}\right)}^{n-1}\right]\right\}-\frac{E_{a}}{RT_{d}}$$

where *T_d_* and α_d_ are the temperature and the conversion degree at the maximum degradation rate, respectively.

A plot of $\ln\left(\frac{q}{T_{d}^{2}}\right)$ against $\frac{1}{T_{d}}$ produces a fitted straight line, and the apparent activation energy, *Ε_a_* is calculated from the slope ($-\frac{E_{a}}{R}$). The advantage of the Kissinger model is that the activation energy can be calculated without any prior knowledge of the thermal degradation reaction mechanism, and *Ε_a_* serves as an indicator of the material’s thermal stability: a high activation energy suggests a high thermal stability.

The relevant Kissinger correlation coefficients (R^2^) and the calculated *Ε_a_*s are presented in the Supplementary Information Table S2, where it should be mentioned that in the multistep decomposition (under air) profiles, the *T_d_* values used for the calculations corresponded to the highest values of the mass loss rate. For the pure grades, the fitting of the data to the Kissinger equation is satisfactory (R^2^: 0.9823–0.9945), with *E_a_* values for the LDPE grades in the range of 303–319 kJ mol^−1^, and for LLDPE slightly lower at 287 kJ mol^−1^. Regarding the FR formulations of both the LDPE grades, the fitting was generally poor, probably because of the multistep character of the decomposition profiles. However, when comparing the data of the satisfactory fittings (R^2^ > 0.95), it can be seen that the *E_a_* values in the LDPE FR-containing formulations are higher compared to those of the pure polymers, e.g., for LDPE-A/FR3 the *E_a_* was found to be 346 kJ mol^−1^ and for LDPE-T/FR4 it was 388 kJ mol^−1^. In the case of the LLDPE FR formulations, the Kissinger model better described the thermo-oxidative degradation kinetics because of the higher R^2^ values (0.914–0.989); LLDPE presented smoother decomposition profiles than the LDPE FR compounds, being similar to those of the pure polymer grades. The general trend for LLDPE is that the FR grades presented higher *E_a_* values compared to the pure polymer, in the range of 308–348 kJ mol^−1^, with FR3 presenting the highest *E_a_* value (348 kJ mol^−1^).

An additional thermal degradation kinetics model, the Coats-Redfern model (Equations (4) and (5) [24]), was examined using the data of thermal (under nitrogen) and thermo-oxidative (under air) decomposition at a heating rate of 10 °C min^−1^. This method can deal with the main degradation region of the TGA curve of the material, and only requires the TGA data at one heating rate to calculate the related reaction order *n*, reaction activation energy *E_a_* and the pre-exponential factor A:

(4) $$n=1,\;\ln\left(-\frac{\ln(1-a)}{T^{2}}\right)=\ln\left[\frac{AR}{qE_{a}}\left(1-\frac{2RT}{E_{a}}\right)\right]-\frac{E_{a}}{RT}$$

(5) $$n\neq 1,\;\ln\left(\frac{1-{\left(1-a\right)}^{1-n}}{T^{2}(1-n)}\right)=\ln\left[\frac{AR}{qE_{a}}\left(1-\frac{2RT}{E_{a}}\right)\right]-\frac{E_{a}}{RT}$$

Generally, the logarithmic term on the right part of the above equations is regarded as constant. The determination of the reaction order n can then be finished by linear fitting of the dependence of the left part of Equations (4) and (5) on –1/T. The n value at the best R^2^ obtained is the real reaction order, and the apparent *E_a_* and the pre-exponential factor A can be calculated. The relevant values are presented in the Tables S3 and S4 in the Supplementary Material. Regarding the pure polymers under nitrogen, the reaction order, n, was calculated close to 1, indicating a first-order degradation kinetics, with a simple degradation reaction mechanism. *E_a_* values under nitrogen (363–537 kJ mol^−1^) were generally found higher compared to those in air (218–413 kJ mol^−1^), in agreement with the aforementioned shifting of the degradation temperatures, *T*_*d*,5%_, and *T_d_*, to lower values under air (Figure S4 in Supplementary Material). However, when comparing the E_a_ values both of the pure and the flame retarded materials, under nitrogen and under air, it can be seen that the pure polymers display *E_a_* values significantly higher than those of the flame retarded materials. Such a result is different from the anticipated behavior, since the incorporation of FRs in the polymer matrix should increase the polymer’s thermal stability and thus an increase in the E_a_ value is expected. It can be herein suggested that the Coats-Redfern method may not be applicable in the case of intumescent flame retardants. That can be attributed to the multistep character of the flame retarded materials’ decomposition profiles [27].

### 3.3. Flammability

**Figure 4:**
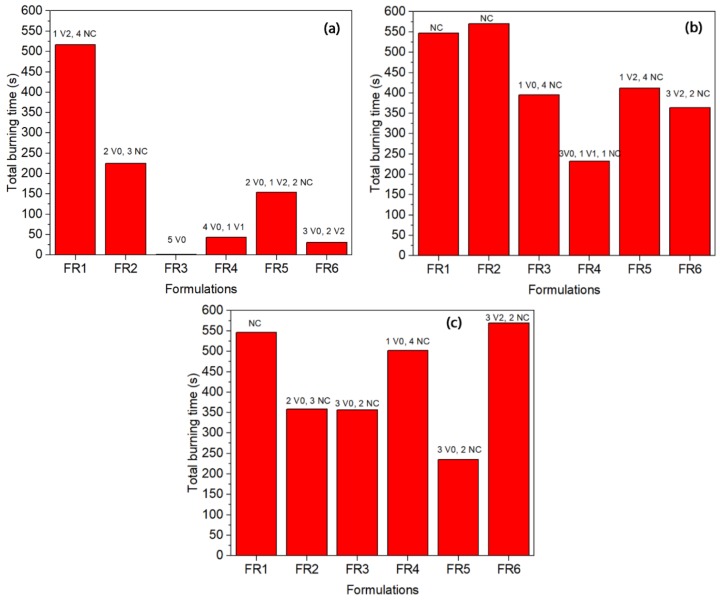
UL 94 V results (total burning time, ranking) for FR-containing polyethylene grades. (**a**) LDPE-A, (**b**) LDPE-T, (**c**) LLDPE.

UL 94 tests are widely used to evaluate the flame resistance of polymers. The results fall into three categories with burning ratings V0, V1, and V2, with V0 corresponding to the highest level of flame resistance. The results of the FR-containing polyethylene grades are given in Figure 4. In all the grades, FR1 presented a low flame resistance since the total burning time was the longest and the samples failed in the UL 94 testing (NC: not classified). It is in agreement with the TGA results where the char yield was low (2%), and this proves that nitrogen-based compounds alone cannot achieve a V0 rating for polyethylene [11]. In fact, the relevant nitrogen-based radical generator is mentioned to perform well when combined with triazine derivatives for polyolefin foams and films [28].

In the case of LDPE-A (Figure 4a), FR3 was proved significantly efficient reaching a UL 94 V0 rating and a low total burning time (1.4 s). FR3 is the mixture of the triazine derivative and APP in a 1:3 ratio of the charring agent to APP with a total loading of 35 wt.%; the mechanism of phosphorus-nitrogen synergism is considered to be provided by the ultimate formation of phosphorous oxynitride, which is a high-temperature resistant material [28]. FR3 performance can be correlated with the TGA results, where it presented the highest onset of thermal degradation for the LDPE-A FR-containing compounds, with an increase in *T_d_* by 16 °C and a char yield of almost 10 ± 1% at 800 °C under nitrogen. FR4 presented the next lower burning time (43.3 s), a performance which can again be correlated with the high char yield in the TGA (14 ± 1% at 800 °C) and an increase in *T_d_* by almost 18 °C. The other formulations (FR2, FR5, FR6) presented poorer results in terms of UL 94 ranking; especially for FR2 it can be said that the commercial mixture of the triazine derivative with APP (PPM Triazine 765) was not as efficient as the FR3-containing grade, where the same mixture was prepared in a ratio CFA:APP = 1:3. The low FR efficiency of FR2 can also be correlated with the zero char yield of the additive alone in the TGA test. The performance of all the examined LDPE-T FRs was poorer than that of LDPE-A. Again, FR3 and FR4 can be considered to be the most promising, but they failed to obtain a safe UL 94 ranking. In the case of LLDPE, FR3 and FR5 presented lower burning times of 356 and 235 s respectively, but again they failed in presenting a safe UL 94 ranking.

**Figure 5:**
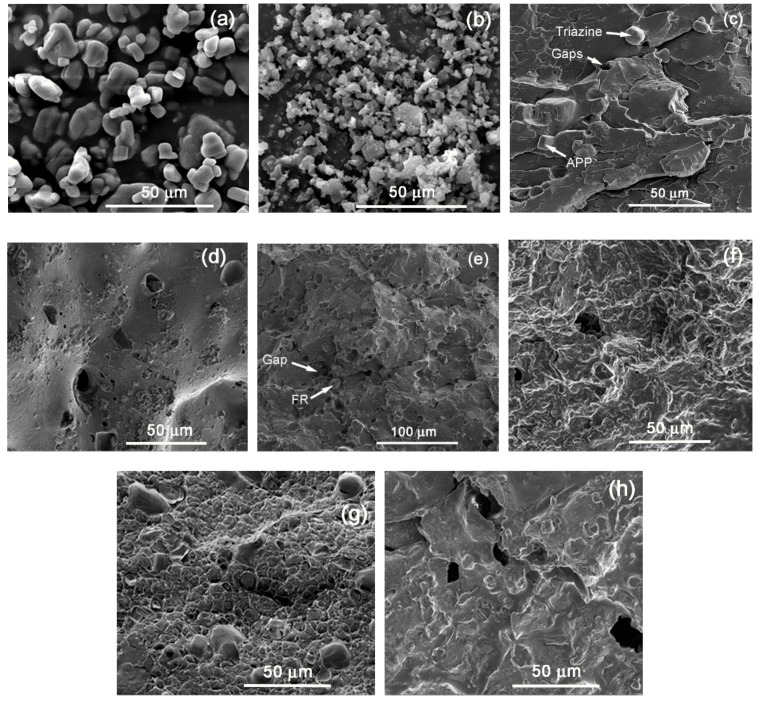
Comparison between FR3-containing fractured surfaces of UL94 bars: (**a**) Neat APP, (**b**) neat Triazine, (**c**) LDPE-A/FR3 prior to UL 94, (**d**) LDPE-A/FR3 char after UL 94, (**e**) LDPE-T/FR3 prior to UL 94, (**f**) LDPE-T/FR3 char after UL 94, (**g**) LLDPE/FR3 prior to UL 94, and (**h**) LLDPE/FR3 char after UL 94.

When comparing the three different polyethylene grades, it is interesting to note that in general, all the FRs presented smaller burning times and higher reproducibility in the case of the LDPE-A compared to the LDPE-T and LLDPE. Inherently, LDPE-A is the least thermally stable polyethylene, as mentioned above, and was found to be more effectively flame retarded through the examined formulations. A possible reason for the poorer performance of the flame retardants in LDPE-T and LLDPE can be the less homogenous dispersion of the additives in the polymer matrix and/or the premature decomposition of the additives, e.g., of APP, due to internal shear during the extrusion and/or the injection step. Therefore, we performed SEM analysis on the fractured cross section surfaces of the UL 94 bars prior to and after the testing (Figure 5). 

Starting with the grade LDPE-A/FR3, which gave the safest V0 ranking result, it can be seen that the additives (APP, Triazine, Figure 5a,b) were well dispersed in the polymer matrix with a small number of aggregates (Figure 5c). The smooth surface of the commercial APP [22] and the shape of Triazine HF can be distinguished in consistence with similar SEM images for APP systems in polyolefins [23]. However, the additives’ incorporation induced some gaps/voids and cavitations, that are a sign of incompatibility and can have a negative impact on the mechanical properties [22]. The gaps/voids can especially be attributed to the premature decomposition of the APP due to internal shear and the release of NH_3_. The char (Figure 5d) for LDPE-A/FR3 looks dense and coherent with an intense foamed structure. Despite the formation of some small holes, a nicely formed intumescent layer is observed which successfully prevented oxygen and heat exchange to penetrate deeper into the material, thus showing the best herein flame retardance performance [20]. 

In LDPE-T/FR3 (Figure 5e) and LLDPE/FR3 (Figure 5g), the additives can also be seen in the polymer matrix, but the surface is less continuous and presents a higher extent of voids, potentially implying a more intense decomposition of APP which will physically impede the formation of a dense and compact protective layer [1] in the subsequent burning test, resulting in formulation failure. The intense decomposition of APP can be correlated with the different melt viscosities and thus with internal shear with respect to the branching type and degree. The processing behavior of LDPE-A and LDPE-T will be different due to the long-chain branching [12,13,14]: LDPE-T presents a higher melt viscosity, since the molecules are strongly entangled with each other due to the comb-like branching, and the shear thinning behavior is stronger at higher rates due to shear-induced partial disentanglement. On the other hand, the LDPE-A molecules with nearly spherical shapes are less entangled and have a lower viscosity. LLDPE has even more voids than LDPE-T, and this can again be attributed to an even higher internal shear. At the shear rates commonly applied during extrusion, LDPE presents increased shear thinning compared to the linear and entangled LLDPE molecules and therefore the melt viscosity of LDPE at higher shear rates is significantly lower than that of the linear resins [12,15]. 

After the UL 94 test, the chars of LDPE-T/FR3 (Figure 5f) and LLDPE/FR3 (Figure 5h) were also investigated by SEM. A different morphology was observed than in the case of LDPE-A/FR3: The char shows some foaming, nevertheless big gaps and/or holes were created which allowed oxygen to penetrate deeper into the material and therefore resulted in poor flame retardancy [20]. This can be the consequence of poor or inhomogenous dispersion of the additives. 

**Figure 6:**
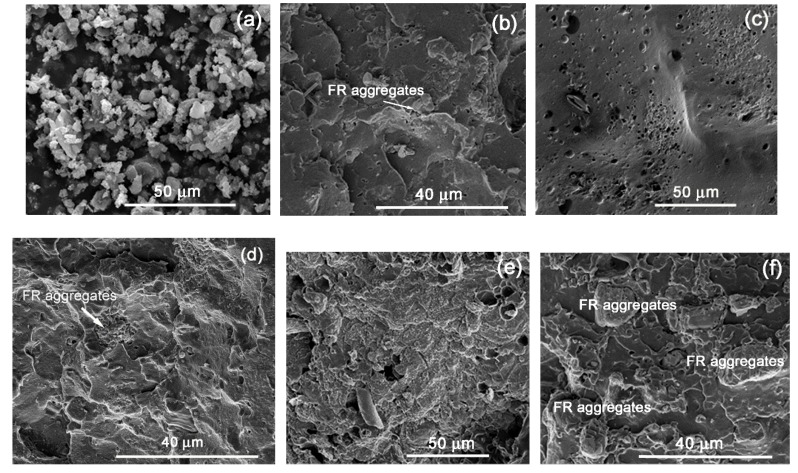

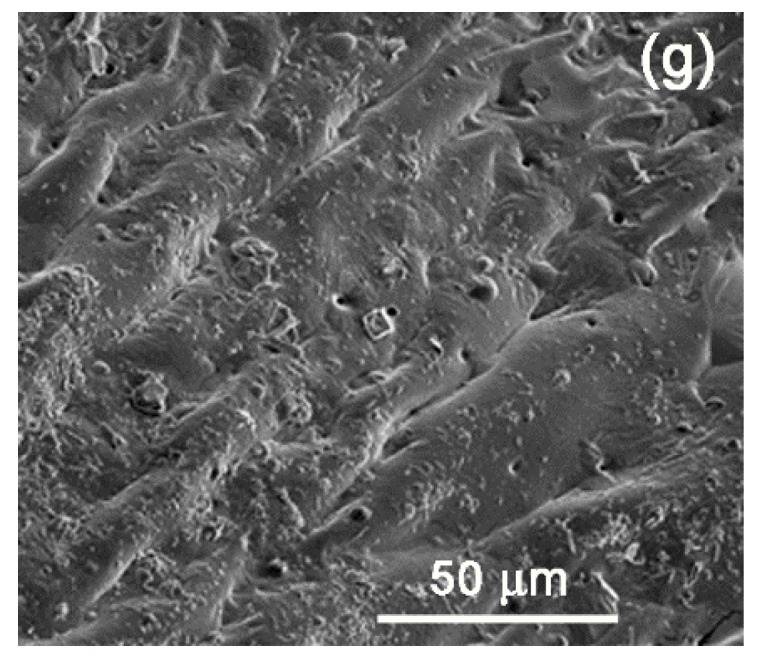
Comparison between FR4-containing fractured surfaces of UL 94 bars: (**a**) Neat Adeka additive, (**b**) LDPE-A/FR4 prior to UL 94, (**c**) LDPE-A/FR4 after UL 94, (**d**) LDPE-T/FR4 prior to UL 94, (**e**) LDPE-T/FR4 after UL 94, (**f**) LLDPE/FR4 prior to UL 94, (**g**) LLDPE/FR4 after UL 94.

On the other hand, in the FR4-containing grades (Figure 6), the voids are significantly less, but in all cases agglomeration of the additive particles is evident, which seems to be the main reason for the inconsistency in the FR results, even in the case of LDPE-A. However, the dispersion problems are more obvious in the case of LLDPE, probably due to the higher melt viscosity. The char quality for all the FR4 grades was also investigated by SEM. Accordingly, LDPE-A/FR4 (Figure 6c) showed a surface similar to that of LDPE-A/FR3, but the swelling of the surface was weaker with numerous tiny holes. This resulted in a lower UL94 classification, nevertheless still four V0 classifications were achieved. On the contrary, in the cases of LDPE-T/FR4 (Figure 6e) and LLDPE/FR4 (Figure 6g), the char morphology was totally different: almost no swelling of the surface and a discontinuous char, resembling the initial morphology of the surfaces prior to the burn test. 

**Figure 7:**
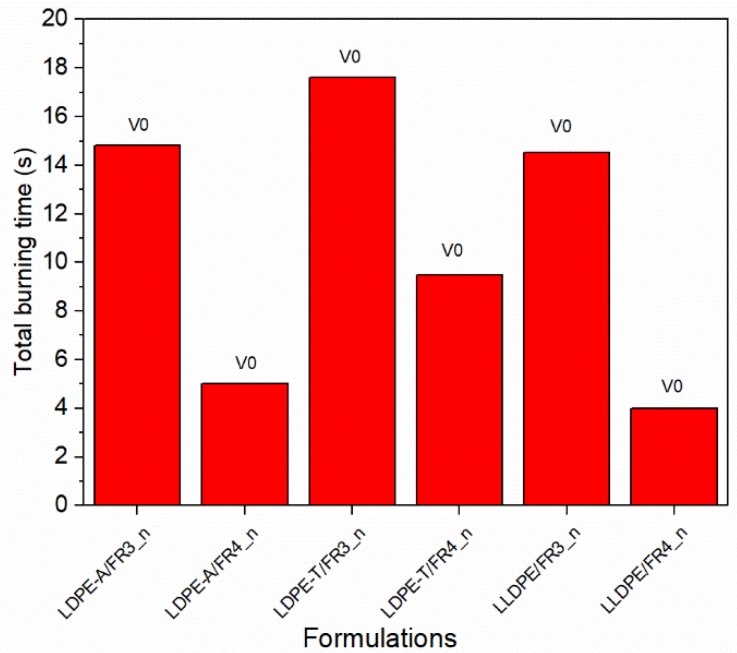
UL94 V results (total burning time, ranking) for the new FR formulations.

To address the dispersion problems and to obtain a more reliable flammability performance, especially for the LDPE-T and LLDPE grades, we tested six (6) additional FR formulations (Table S5 in the Supplementary Information) based on the most promising ones (FR3 and FR4) using compression molding to manufacture the UL94 bars instead of injection molding, so as to dissipate the melt viscosity issues. The new compounds presented stable V0 classification with no dripping and total burning times ranging from 4.0 to 17.6 s (Figure 7), revealing a reliable FR performance due to the higher UL94 bars’ homogeneity.

### 3.4. Mechanical and Rheological (MFR) Properties

Table 2 summarizes the rheological and mechanical properties of the three pure polyethylenes and of the most promising FR formulations (FR3 and FR4 compounds). The processability of the FR-containing grades can be roughly assessed based on the MFR values. Regarding the LDPE grades, the FR3 formulation did not greatly influence the MFR of the pure polymers, while FR4 induced a decrease by more than 50%, indicating higher melt viscosities. In the case of LLDPE, both formulations (FR3 and FR4) resulted in MFR decrease by more than 40%.

Regarding the mechanical properties, pure LLDPE has higher values of elongation at break than LDPE, because of the difference in branching [19]. When adding the FRs, it can be generally said that the tensile and the impact strength were not significantly affected, despite the high loadings (FR3: 35 wt.%, FR4: 30 wt.%). More specifically, in LDPE-A, the tensile strength and the elongation at break decreased by 27–33% (FR3) and 23–34% (FR4), along with an increase of the Young’s modulus (E) by 27 and 15% respectively; the corresponding impact strength remained constant at ca. 20 kJ m^−2^. The relevant changes can be considered to be lower than or comparable to the values mentioned in literature (>25% change for tensile strength) [1,21] for similar loadings of halogen-free flame retardants. Keeping in mind that LDPE-A/FR3 is the most promising formulation in terms of safe UL94 V0 ranking, it is concluded that it can also satisfy the requirements in terms of good mechanical properties.

The LDPE-T and LLDPE formulations presented changes in the tensile properties compared to the pure grades similar to those of LDPE-A, despite the poorer distribution and dispersion of the additives in the polymer matrix, as discussed above based on the SEM analysis. In fact, the most significant change was observed in the Young’s modulus of LDPE-T: E increased with ca.50% for the FR3 and FR4 compared to the pure grades, with FR4 showing the highest stiffness in both grades. 

### 3.5. Melting Behavior

The melting behavior of the most promising FR formulations (FR3 and FR4 compounds) was examined in order to identify the effect of the flame retardants on the melting point and the crystallinity of the polyethylene grades (Table 3). 

Pure LDPE-T was found to crystallize earlier (at higher *T*_c_) than LDPE-A, presenting a higher mass fraction crystallinity and a higher melting point during the 2nd heating. The crystal density in LDPE-A was obviously lowered by the incorporation of chain imperfections into the crystal lattice and resulted in lower values of x_c_ [12]. Autoclave resins tend to have better see-through clarity due to the smaller spherulites formed during the crystallization process [12]. The crystals in LLDPE, which exhibits a higher density fraction with minimal branching, are thicker than those in LDPE [15], and therefore the determined *T*_m_ values were found at 122 °C, ca. 10 °C higher than those of the two LDPEs. The minimal branching of LLDPE also permits faster melt crystallization, so the *T*_c_ value is increased (ca. 105 °C for LLDPE vs. 94 and 96 °C for LDPE-A and LDPE-T respectively). 

In general, the incorporation of the flame retardants (FR3, FR4) at the specific loadings (30 and 35 wt.%) did not significantly change the thermal properties of the polyethylene grades, implying limited action of the additives as nucleating agents: A slight increase in the melting point by almost 2 °C was found for all three PE grades, while in the case of FR3, an increase in the mass fraction crystallinity was also observed without a significant change in the *T*_c_ temperature for LDPE-A and LDPE-T. APP was found to act as an effective nucleating agent in the case of PP at loadings of 30% wt. [29,30], so its high content in FR3 (26.25 wt.%) may explain the higher attained crystallinity in all three the PE grades compared to FR4. Finally, in the case of the most easily crystallizing LLDPE, the nucleating role of the additives becomes more noticeable, since along with the increase in *x_c_* (2nd heating), the melt crystallization upon cooling also occurred earlier, i.e., at a noticeable higher *T*_c_.

## 4. Conclusions

The performance of commercial halogen-free flame retardants was investigated for two grades of low-density polyethylene (LPDE) and one linear low-density polyethylene (LLPDE). An FR formulation of a triazine derivative and ammonium polyphosphate at a ratio of 1:3 and a total loading of 35 wt.% was found to be the most efficient for the low-density polyethylene produced in an autoclave reactor, achieving a UL 94 V0 ranking and upgrading the thermal stability of the polymer: the thermal degradation temperature was increased by more than 15 °C, along with a char residue which reached 10 ± 1% at 800 °C. Accordingly, the kinetics of the thermo-oxidative decomposition based on the Kissinger model showed an increase in the activation energy, which reached 346 kJ mol^−1^ vs. 303 kJ mol^−1^ for the pure grade. The thermal, rheological (MFR) and mechanical properties did not significantly change for this most promising LDPE-A/FR3 formulation. On the other hand, in the case of the other polyethylene grades (LDPE from the tubular reactor and LLDPE), the performance of the specific formulation was poorer and correlated with the different rheological properties: The LDPE-T and LLDPE molecules are strongly entangled due to the extensibility of the long main-chain, increasing the melt viscosity and the internal shear, which in turn resulted in poor FRs’ dispersion and/or premature thermal decomposition of the ammonium polyphosphate and thus less FR activity. 

## Figures and Tables

**Table 1:** Composition of FR formulations in wt.% for polyethylene grades. All formulations contained 0.05 wt.% calcium stearate.

FRs	Flamestab NOR116	PPM Triazine 765	PPM Triazine HF	Charmor DP40	Charmor PP100	Exolit AP422	ADK Stab FP2200	Total FR (wt.%)	CFA:APP
FR1	5							5	
FR2		30						30	
FR3			8.75			26.25		35	1:3
FR4 *							30	30	
FR5				7		21	7	35	1:1.5
FR6					7	21	7	35	1:1.5

* 0.1 wt.% calcium stearate.

**Table 2:** Rheological (MFR) and mechanical properties of pure polyethylene grades and selective FR polyolefin formulations.

FRs	MFR (g/10 min)	Tensile Strength (MPa)	Elongation at Break (%)	Young’s Modulus (MPa)	Izod Impact Strength (kJ m^−2^)
**LDPE-A**					
Pure	0.28 ± 0.01	18.1 ± 0.2	145 ± 6	133 ± 6	19.1 ± 4.9
FR3	0.22 ± 0.01	13.2 ± 0.6	96.7 ± 2.7	169.1 ± 13.8	20.5 ± 2.5
FR4	0.11 ± 0.01	13.9 ± 0.4	95.0 ± 6.0	152.6 ± 12.5	21.5 ± 0.7
**LDPE-T**					
Pure	0.28 ± 0.02	17.8 ± 0.2	130 ± 3	119 ± 3	17.3 ± 2.0
FR3	0.25 ± 0.02	12.8 ± 0.4	92.7 ± 14.5	178.6 ± 11.6	18.9 ± 1.4
FR4	0.17 ± 0.01	15.5 ± 0.4	99.1 ± 24.1	183.9 ± 15.6	20.4 ± 0.9
**LLDPE**					
Pure	1.01 ± 0.01	13.1 ± 0.3	420.8 ± 26.1	130.4 ± 4.5	23.4 ± 2.1
FR3	0.56 ± 0.04	14.4 ± 0.7	315.6 ± 59.7	140.7 ± 6.3	21.9 ± 2.2
FR4	0.58 ± 0.05	13.3 ± 0.2	219.7 ± 42.0	157.5 ± 14.4	21.8 ± 2.0

**Table 3:** Thermal properties of pure polyethylene grades and selective FR polyolefin formulations.

FRs	Cooling			2nd Heating		
	*T*_c_ (°C)	Δ*H*_c_ (J g^−1^)	*x_c_* (%)	*T*_m_ (°C)	Δ*H*_m_ (J g^−1^)	x_c_ (%)
**LDPE-A**						
Pure	94.4 ± 0.0	90.7 ± 6.7	31.0 ± 2.3	110.1 ± 0.0	91.3 ± 6.8	31.2 ± 2.3
FR3	93.2 ± 1.4	108.5 ± 4.4	37.0 ± 1.5	112.6 ± 0.8	102.9 ± 7.6	35.1 ± 2.6
FR4	91.7 ± 1.0	93.1 ± 6.9	31.8 ± 2.4	114.0 ± 1.5	93.7 ± 6.5	32.0 ± 2.2
**LDPE-T**						
Pure	96.1 ± 0.1	96.9 ± 0.8	33.1 ± 0.3	112.5 ± 0.2	97.4 ± 0.1	33.2 ± 0.0
FR3	95.0 ± 0.4	108.3 ± 4.8	37.0 ± 1.6	114.2 ± 0.7	109.2 ± 6.1	37.3 ± 2.1
FR4	95.4 ± 0.0	98.7 ± 7.3	33.7 ± 2.5	114.6 ± 0.1	98.6 ± 6.5	33.6 ± 2.2
**LLDPE**						
Pure	104.9 ± 0.6	91.9 ± 2.5	31.4 ± 0.9	122.0 ± 0.1	94.1 ± 0.7	32.1 ± 0.2
FR3	107.1 ± 0.1	99.4 ± 2.4	33.9 ± 0.8	124.0 ± 0.1	101.9 ± 2.5	34.8 ± 0.8
FR4	108.6 ± 0.1	86.4 ±10.9	29.5 ± 3.7	124.4 ± 0.2	87.9 ± 10.6	30.0 ± 3.6

## Supplementary Materials

The following are available online at https://www.mdpi.com/2073-4360/11/9/1479/s1, Figure S1: Conformation plot for (a) LLDPE, (b) LDPE-A, and (c) LDPE-T. The green line is a linear fit of the data; Figure S2: (a) TGA curves of the pure polymers (LDPE-A, LDPE-T, LLDPE) under air and nitrogen atmosphere, (b) Correlation between the onset of thermal degradation (*T*_*d*,5%_) and the temperature at the maximum rate of weight loss (*T_d_*) for the pure polymers under air and nitrogen atmosphere; Figure S3: TGA curves of pure FR additives under nitrogen atmosphere; Figure S4: Correlation between the onset of thermal degradation (*T*_*d*,5%_) vs. the temperature at the maximum rate of weight loss (*T_d_*) for the FR-containing compounds under (a) nitrogen and (b) air atmosphere TGA; Table S1: Characteristics of the examined halogen-free flame retardants (FRs); Table S2: Activation energies (*E_a_*) and correlation coefficients (R^2^) of thermo-oxidative degradation (TGA under air) of FR polyolefin systems according to the Kissinger model; Table S3: The kinetic parameters (reaction order (n), activation energy (*E_a_*), pre-exponential factor (A)) of FR polyolefin systems using the Coats-Redfern method (TGA under nitrogen); Table S4: The kinetic parameters (reaction order (n), activation energy (*E_a_*), pre-exponential factor (A)) of the FR polyolefin systems using the Coats-Redfern method (TGA under air); Table S5: Composition of the new FR formulations in wt% for the polyethylene grades.

## References

[1] Ba M., Liang B., Wang C. (2017). Synthesis and characterization of a novel charring agent and its application in intumescent flame-retardant polyethylene system. Fiber Polym..

[2] Aubert M., Tirri M., Wilen C.-E., Francois-Heude A., Pfaendner R., Hoppe H., Roth M. (2012). Versatile bis(1-alkoxy-2,2,6,6-tetramethylpiperidin-4-yl) diazenes (AZONORs) and related structures and their utilization as flame retardants in polypropylene, low density polyethylene and high impact polystyrene. Polym. Degrad. Stab..

[3] Pfaendner R., Spalding M.A., Chatterjee A.M. (2017). Flame retardants for polyethylene. Handbook of Industrial Polyethylene and Technology.

[4] Papaspyrides C., Kiliaris P. (2014). Polymer Green Flame Retardants.

[5] Pinfa Phosphorus, Inorganic & Nitrogen Flame Retardants Association. www.pinfa.eu.

[6] Liang B., Hong X., Zhu M., Gao C., Wang C., Tsubaki N. (2015). Synthesis of novel intumescent flame retardant containing phosphorus, nitrogen and boron and its application in polyethylene. Polym. Bull..

[7] Nie S., Zhang M., Yuan S., Dai G., Hong N., Song L., Hu Y., Liu X. (2012). Thermal and flame-retardant properties of novel intumescent flame-retardant low-density polyethylene (LDPE) composites. J. Therm. Anal. Calorim..

[8] Nie S., Hu Y., Song L., He Q., Yang D., Chen H. (2008). Synergistic effect between a char forming agent (CFA) and microencapsulated ammonium polyphosphate on the thermal and flame-retardant properties of polypropylene. Polym. Adv. Technol..

[9] Hu X., Li Y. (2004). Synergistic effect of the charring agent on the thermal and flame-retardant properties of polyethylene. Macromol. Mater. Eng..

[10] Shao Z., Deng C., Tan Y., Chen M., Chen L., Wang Y. (2014). Flame retardation of polypropylene via a novel intumescent flame retardant: Ethylenediamine-modified ammonium polyphosphate. Polym. Degrad. Stab..

[11] Liao S., Deng C., Huang S., Cao J., Wang Y. (2016). An efficient halogen-free flame retardant for polyethylene: piperazine-modified ammonium polyphosphates with different structures. Chin. J. Polym. Sci..

[12] Maraschin N. (1989). Ethylene polymers LDPE. Encyclopedia of Polymer Science and Technology.

[13] Kuhn R., Kromer H. (1982). Structures and properties of different low density polyethylenes. Colloid. Polym. Sci..

[14] Pandey G., Singh B., Kulshreshtha A. (1990). Morphological characterization of autoclave and tubular LDPE by high temperature IR spectroscopy. Polym. Test..

[15] Simpson D., Vaughan G. (2002). Ethylene polymers, LLDPE. Encyclopedia of Polymer Science and Technology.

[16] Molefi J., Luyt A., Krupa I. (2010). Comparison of LDPE, LLDPE and HDPE as matrices for phase change materials based on a soft Fischer-Tropsch paraffin wax. Thermochim. Acta..

[17] Billingham N. (2002). Degradation. Encyclopedia of Polymer Science and Technology.

[18] Al-Malaika S. (2001). Stabilization. Encyclopedia of Polymer Science and Technology.

[19] Luyt A.S.L., Molefi J., Krump H. (2006). Thermal, mechanical and electrical properties of copper powder filled low-density and linear low-density polyethylene composites. Polym. Degrad. Stab..

[20] Makhlouf G., Hassan M., Nour M., Abdel-Monem Y., Abdelkhalik A. (2017). Evaluation of fire performance of linear low-density polyethylene containing novel intumescent flame-retardant. J. Therm. Anal. Calorim..

[21] Xie R., Qu B. (2001). Synergistic effects of expandable graphite with some halogen-free flame retardants in polyolefin blends. Polym. Degrad. Stab..

[22] Yu T., Tuerhongjiang T., Sheng C., Li Y. (2017). Phosphorus-containing diacid and its application in jute/poly(lactic acid) composites: Mechanical, thermal and flammability properties. Compos. Part A Appl. Sci. Manuf..

[23] Shao Z., Deng C., Tan Y., Chen M., Chen L., Wang Y. (2014). An efficient mono-component polymeric intumescent flame retardant for polypropylene: Preparation and application. ACS Appl. Mater. Interfaces.

[24] Chen Y., Wang Q. (2007). Thermal oxidative degradation kinetics of flame-retarded polypropylene with intumescent flame-retardant master batches in situ prepared in twin-screw extruder. Polym. Degrad. Stab..

[25] Lecouvet B., Bourbigot S., Sclavons M., Bailly C. (2012). Kinetics of the thermal and thermo-oxidative degradation of polypropylene/halloysite nanocomposites. Polym. Degrad. Stab..

[26] Yee T., Lin O., Bindumadhavan K., Doong R. (2017). Unveiling the thermal kinetics and scissoring mechanism of neolatry polyethylene/reduced graphite oxide nanocomposites. J. Anal. Appl. Pyrolysis.

[27] Ebrahimi-Kahrizsangi R., Abbasi M.-H. (2008). Evaluation of reliability of Coats-Redfern method for kinetic analysis of non-isothermal TGA. Trans. Nonferr. MMet. Soc. China.

[28] Kaul B. (2016). PPM triazines lightweight organo-polymeric universal fire and flame-retardant synergists. Rubber Fiber Plast..

[29] Wu K., Zhang Y., Hu W., Lian J., Hu Y. (2013). Influence of ammonium polyphosphate microencapsulation on flame retardancy, thermal degradation and crystal structure of polypropylene composite. Compos. Sci. Technol..

[30] Lu M., Zhang S., Yu D. (2004). Study on poly(propylene)/Ammonium polyphosphate composites modified by ethylene-1-octene copolymer grafted with glycidyl methacrylate. J. Appl. Polym. Sci..

