# Supplementary material for: Halogen-Free Flame-Retardant Compounds. Thermal Decomposition and Flammability Behavior for Alternative Polyethylene Grades
Source: Polymers (Basel). 2019 Sep 10;11(9):1479. doi: 10.3390/polym11091479 (PMC6780580; doi:10.3390/polym11091479)
Supplement: Supplementary file 1 [file polymers-11-01479-s001.pdf]

Supplementary Materials: Halogen-Free Flame-Retardant Compounds. Thermal Decomposition and Flammability Behavior for Alternative Polyethylene Grades

Adriaan Stephanus Luyt, Sarah Shahid Malik, Soumia Abderrazak Gasmi, Athanasios Porfyris, Anna Andronopoulou, Dimitrios Korres, Stamatina Vouyiouka, Michael Grosshauser, Rudolf Pfaendner, Robert Brüll, Constantine Papaspyrides

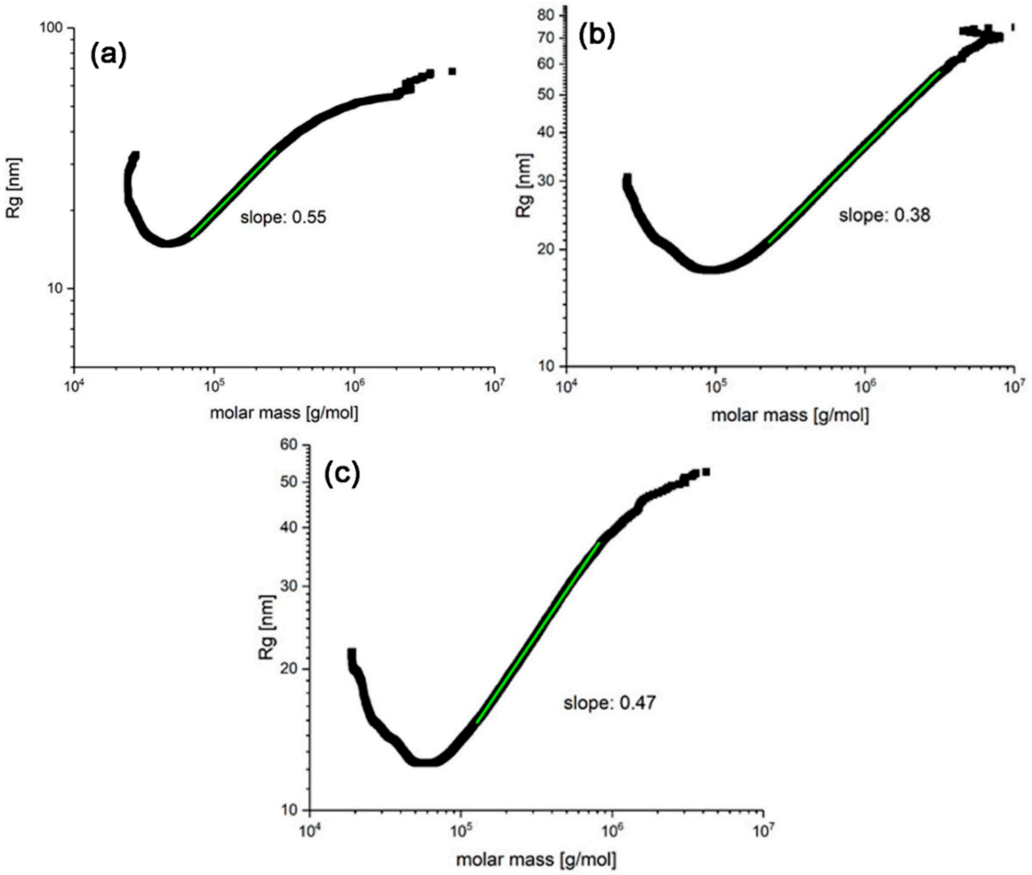

Figure S1. Conformation plot for (a) LLDPE, (b) LDPE-A, and (c) LDPE-T. The green line is a linear fit of the data.

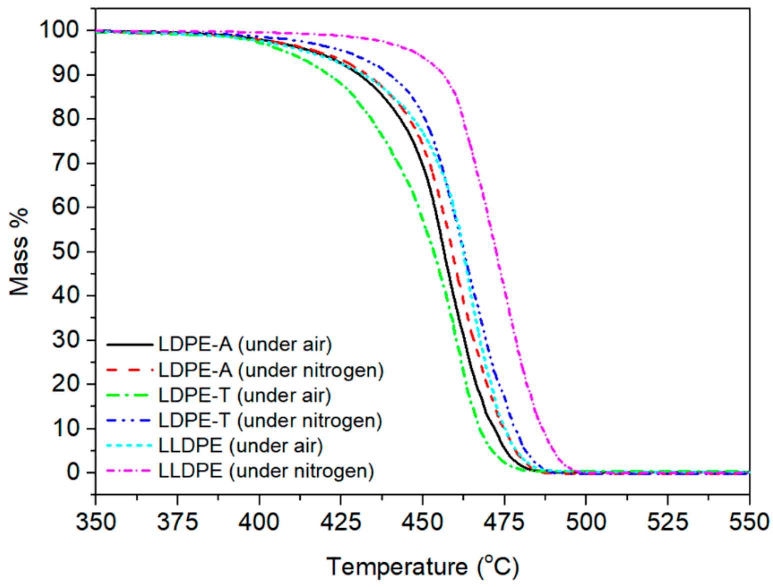

(a)

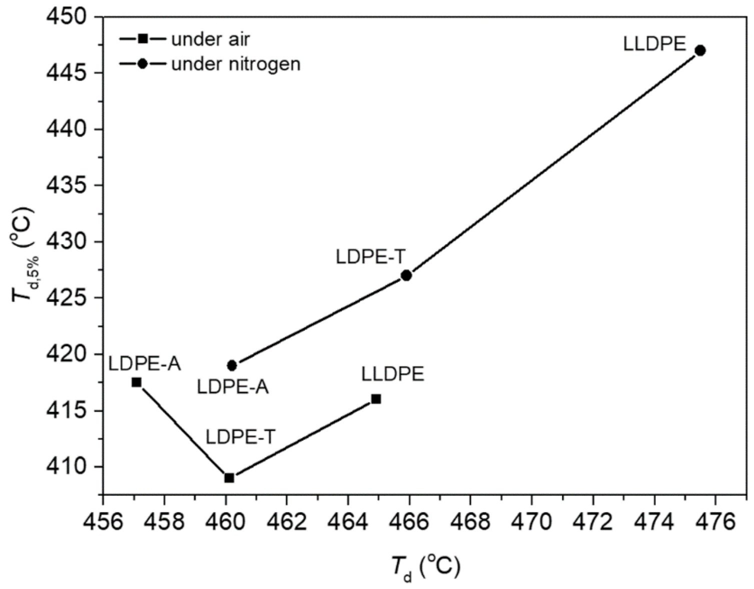

(b)

Figure S2. (a) TGA curves of the pure polymers (LDPE-A, LDPE-T, LLDPE) under air and nitrogen atmosphere, (b) Correlation between the onset of thermal degradation ($T_{d,5\%}$) and the temperature at the maximum rate of weight loss (T_d) for the pure polymers under air and nitrogen atmosphere.

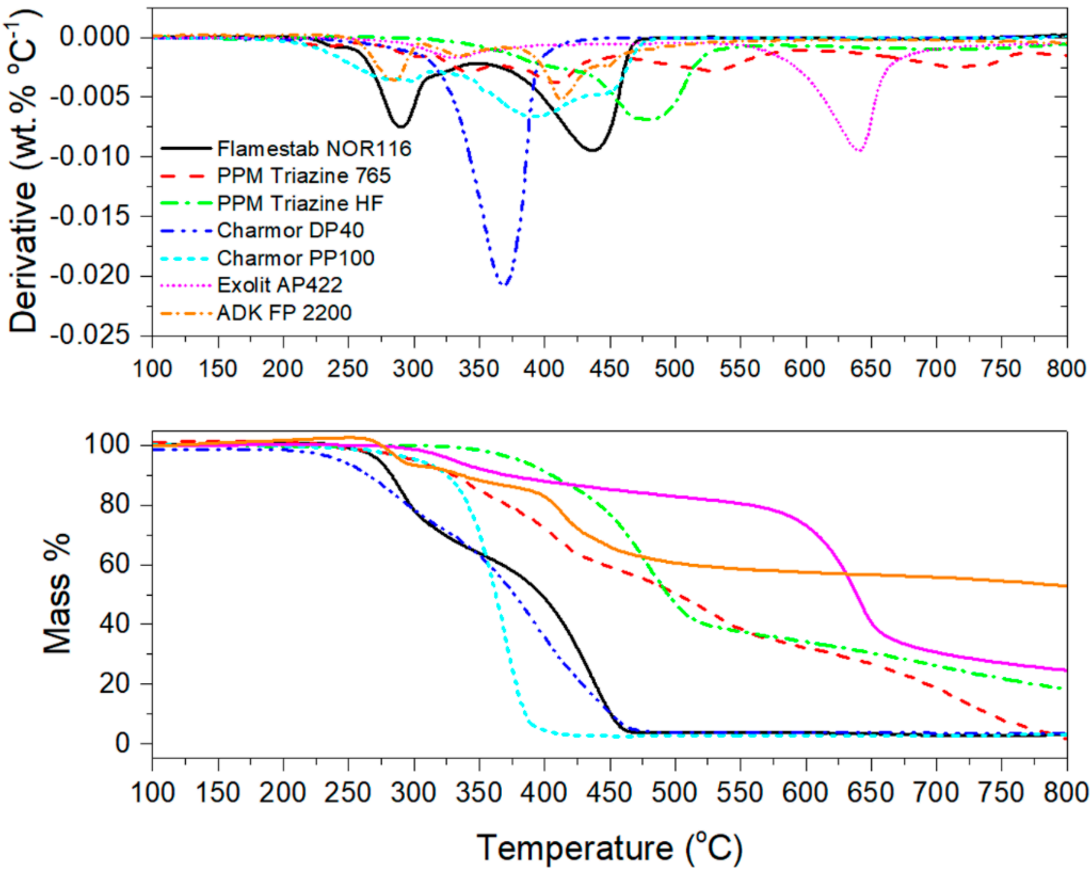

Figure S3. TGA curves of pure FR additives under nitrogen atmosphere.

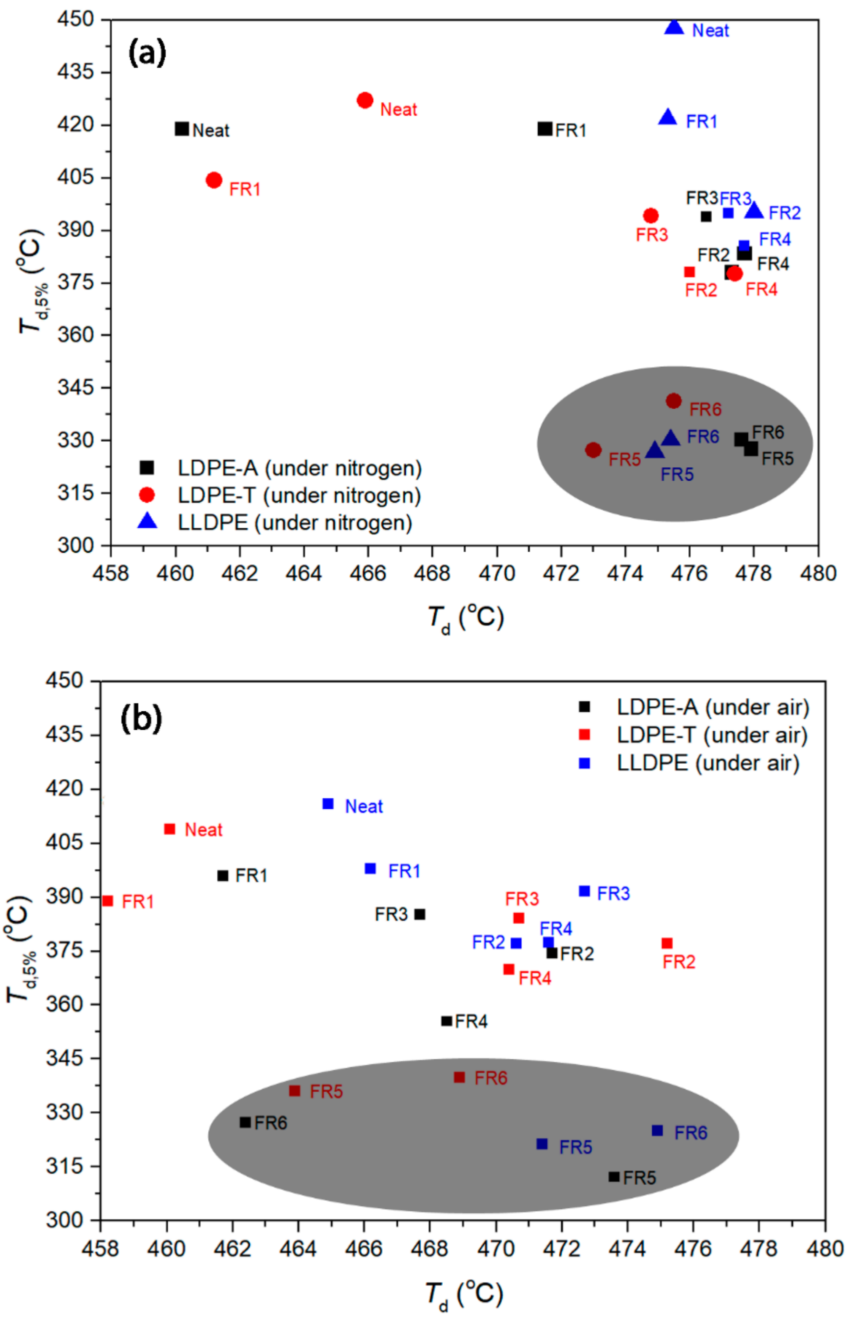

Figure S4. Correlation between the onset of thermal degradation ($T_{d,5\%}$) vs. the temperature at the maximum rate of weight loss (T_d) for the FR-containing compounds under (a) nitrogen and (b) air atmosphere TGA.

Table S1. Characteristics of the examined halogen-free flame retardants (FRs).

FR Product (Company)	Composition	MW (g mol^{-1})	Melting (°C)	TGA weight loss (%)
Flamestab NOR 116<br>(BASF)	Monomeric N-alkoxy hindered amine<br>(triazine derivative)<br>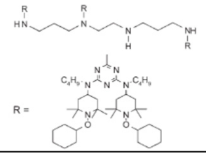	2261	108-123	1% (260 °C)<br>10 % (285 °C)
MCA® PPM Triazine 765<br><br>MCA® PPM Triazine HF<br>(MCA Technologies GmbH)	Ammonium polyphosphate (APP) + poly-[2, 4-(piperazine-1, 4-yl)-6-(morpholine-4-yl)-1, 3, 5-triazine]/Piperazin (MCA® PPM triazine HF)<br>poly-[2, 4-(piperazine-1, 4-yl)-6-(morpholine-4-yl)-1, 3, 5-triazine]/Piperazin<br>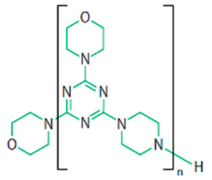	>2500	Infusible<br>(>290°C)	<2 % up to 300 °C
Exolit AP422<br>(Clariant)	Ammonium polyphosphate (APP)<br>$[NH_4PO_3]_n$<br>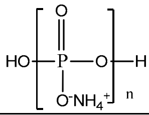	n>1000		5 % (350 °C)<br>10 % (450 °C)<br>20 % (550 °C)
ADK Stab FP-2200<br>(Adeka)	Nitrogen-phosphorus FR (blend)		Not observed<br>(< 270 °C)	1% (260 °C)<br>5% (285 °C)
Charmor DP40<br>Charmor PP100<br>(Perstorp)	Pentaerythritol derivative, dimer<br>Pentaerythritol derivative, polymer		222 °C<br>160-180 °C	Onset 330 °C<br>240 °C

Table S2. Activation energies (E_a) and correlation coefficients (R^2) of thermo-oxidative degradation (TGA under air) of FR polyolefin systems according to the Kissinger model.

	E_a (kJ mol^{-1})	R^2
**LDPE-A**	303	0.9823
**LDPE-A/FR1**	353	0.9714
**LDPE-A/FR2**	211	0.9167
**LDPE-A/FR3**	346	0.9874
**LDPE-A/FR4**	498	0.6825
**LDPE-A/FR5**	292	0.8607
**LDPE-A/FR6**	391	0.7822
**LDPE-T**	319	0.9894
**LDPE-T/FR1**	432	0.9977
**LDPE-T/FR2**	397	0.7877
**LDPE-T/FR3**	262	0.8391
**LDPE-T/FR4**	388	0.9923
**LDPE-T/FR5**	282	0.6190
**LDPE-T/FR6**	461	0.7612
**LLDPE**	287	0.9945
**LLDPE/FR1**	324	0.9538
**LLDPE/FR2**	274	0.9739
**LLDPE/FR3**	348	0.9728
**LLDPE/FR4**	308	0.9890
**LLDPE/FR5**	328	0.9139
**LLDPE/FR6**	274	0.9873

Table S3. The kinetic parameters (reaction order (n), activation energy (E_a), pre-exponential factor (A)) of FR polyolefin systems using the Coats-Redfern method (TGA under nitrogen).

	n	E_a (kJ mol^{-1})	A	R^2
**LDPE-A**	1.1	363	5.135×10^{22}	0.9968
**LDPE-A/FR1**	0.9	328	5.713×10^{19}	0.9944
**LDPE-A/FR2**	0.4	214	3.070×10^{11}	0.9953
**LDPE-A/FR3**	0.7	218	6.244×10^{11}	0.9974
**LDPE-A/FR4**	0.2	185	2.488×10^{9}	0.9991
**LDPE-A/FR5**	1.0	249	1.076×10^{14}	0.9969
**LDPE-A/FR6**	0.9	245	5.766×10^{13}	0.9966
**LDPE-T**	1.2	408	6.266×10^{25}	0.9991
**LDPE-T/FR1**	1.0	340	1.071×10^{21}	0.9956
**LDPE-T/FR2**	0.3	159	3.297×10^{7}	0.9940
**LDPE-T/FR3**	0.4	157	2.429×10^{7}	0.9937
**LDPE-T/FR4**	0.1	148	4.703×10^{6}	0.9943
**LDPE-T/FR5**	0.4	156	2.187×10^{7}	0.9946
**LDPE-T/FR6**	0.6	205	8.135×10^{10}	0.9981
**LLDPE**	1.5	537	4.240×10^{34}	0.9989
**LLDPE/FR1**	0.9	327	4.600×10^{19}	0.9996
**LLDPE/FR2**	0.6	229	3.826×10^{12}	0.9984
**LLDPE/FR3**	0.5	184	2.083×10^{9}	0.9953
**LLDPE/FR4**	0.2	168	1.452×10^{8}	0.9953
**LLDPE/FR5**	0.5	188	4.279×10^{9}	0.9971
**LLDPE/FR6**	0.6	209	1.373×10^{11}	0.9951

Table S4. The kinetic parameters (reaction order (n), activation energy (E_a), pre-exponential factor (A)) of the FR polyolefin systems using the Coats-Redfern method (TGA under air).

	n	E_a (kJ mol^{-1})	A	R^2
**LDPE-A**	1.3	413	2.782×10^{26}	0.9972
**LDPE-A/FR1**	1.3	337	5.291×10^{20}	0.9979
**LDPE-A/FR2**	0.4	128	2.521×10^{5}	0.9935
**LDPE-A/FR3**	0.5	120	6.542×10^{4}	0.9982
**LDPE-A/FR4**	0.3	127	2.099×10^{5}	0.9965
**LDPE-A/FR5**	0.9	158	4.091×10^{7}	0.9982
**LDPE-A/FR6**	0.4	170	1.947×10^{8}	0.9978
**LDPE-T**	0.4	218	1.358×10^{12}	0.9973
**LDPE-T/FR1**	0.4	207	2.107×10^{11}	0.9968
**LDPE-T/FR2**	0.4	97	1.245×10^{3}	0.9978
**LDPE-T/FR3**	0.1	89	2.814×10^{2}	0.9985
**LDPE-T/FR4**	0.5	135	9.051×10^{5}	0.9980
**LDPE-T/FR5**	1.1	277	9.417×10^{15}	0.9945
**LDPE-T/FR6**	0.2	167	9.916×10^{7}	0.9930
**LLDPE**	0.8	347	2.281×10^{21}	0.9913
**LLDPE/FR1**	1.1	298	9.826×10^{17}	0.9977
**LLDPE/FR2**	0.3	101	2.527×10^{3}	0.9908
**LLDPE/FR3**	0.1	109	8.588×10^{3}	0.9896
**LLDPE/FR4**	0.1	135	7.127×10^{5}	0.9964
**LLDPE/FR5**	0.5	185	2.951×10^{9}	0.9972
**LLDPE/FR6**	0.5	189	5.176×10^{9}	0.9959

Table S5. Composition of the new FR formulations in wt% for the polyethylene grades.

	**PPM Triazine HF**	**Exolit AP422**	**ADK Stab FP2200**	**Total FR (% wt)**	**CFA:APP**
LDPE-A/FR3_n	8.75	26.25		35	1:3
LDPE-A/FR4_n			35	35	
LDPE-T/FR3_n	14	21		35	2:3
LDPE-T/FR4_n			30	30	
LLDPE/FR3_n	8.75	26.25		35	1:3
LLDPE/FR4_n			40	40	
